# Benchmarking pK_a _prediction

**DOI:** 10.1186/1471-2091-7-18

**Published:** 2006-06-02

**Authors:** Matthew N Davies, Christopher P Toseland, David S Moss, Darren R Flower

**Affiliations:** 1 Edward Jenner Institute for Vaccine Research, Compton, Berkshire, RG20 7NN, UK; 2 School of Crystallography, Birkbeck College, Malet Street, London WC1E 7HX, UK

## Abstract

**Background:**

pK_a _values are a measure of the protonation of ionizable groups in proteins. Ionizable groups are involved in intra-protein, protein-solvent and protein-ligand interactions as well as solubility, protein folding and catalytic activity. The pK_a _shift of a group from its intrinsic value is determined by the perturbation of the residue by the environment and can be calculated from three-dimensional structural data.

**Results:**

Here we use a large dataset of experimentally-determined pK_a_s to analyse the performance of different prediction techniques. Our work provides a benchmark of available software implementations: MCCE, MEAD, PROPKA and UHBD. Combinatorial and regression analysis is also used in an attempt to find a consensus approach towards pK_a _prediction. The tendency of individual programs to over- or underpredict the pK_a _value is related to the underlying methodology of the individual programs.

**Conclusion:**

Overall, PROPKA is more accurate than the other three programs. Key to developing accurate predictive software will be a complete sampling of conformations accessible to protein structures.

## Background

A proper understanding of protein pK_a _values is essential to a proper understanding of pH-dependent characteristics of protein function. If the pK_a _of a particular group is known then one can determine its protonation state at a given pH, helping to determine several important properties including protein solubility, protein folding and catalytic activity. Knowledge of the pK_a _values of the residues of an active site can help to identify the reaction mechanism of an enzyme or aid in the interpretation of experimental results [1-4]. The pK_a _value is -log_10_(K_a_) where K_a_, is the ionization constant, a measure of a titratable group's ability to donate a proton:

Ka=[H+][A−][HA]     (1)
 MathType@MTEF@5@5@+=feaafiart1ev1aaatCvAUfKttLearuWrP9MDH5MBPbIqV92AaeXatLxBI9gBaebbnrfifHhDYfgasaacH8akY=wiFfYdH8Gipec8Eeeu0xXdbba9frFj0=OqFfea0dXdd9vqai=hGuQ8kuc9pgc9s8qqaq=dirpe0xb9q8qiLsFr0=vr0=vr0dc8meaabaqaciaacaGaaeqabaqabeGadaaakeaacqWGlbWsdaWgaaWcbaGaemyyaegabeaakiabg2da9maalaaabaGaei4waSLaemisaG0aaWbaaSqabeaacqGHRaWkaaGccqGGDbqxcqGGBbWwcqWGbbqqdaahaaWcbeqaaiabgkHiTaaakiabc2faDbqaaiabcUfaBjabdIeaijabdgeabjabc2faDbaacaWLjaGaaCzcamaabmaabaGaeGymaedacaGLOaGaayzkaaaaaa@4224@

The pK_a _value is therefore equal to the pH when there is an equal concentration of the protonated and deprotonated groups in solution. Each residue with a titratable group has a model or 'intrinsic' pK_a _value, defined as the pK_a _value when all the other groups are fixed in their neutral state. Ionizable groups may be divided into acidic, which are neutral in their protonated state, and basic, which are positively charged in their protonated state. The protonated and the non-protonated forms of a residue can be very different chemically. In the case of His, the protonated form is hydrophilic and positively charged while the non-protonated form has a hydrophobic and aromatic character. Consequently the nature of the interaction made by an ionizable group may differ significantly at a pH above or below the pK_a_.

Table 1 shows the intrinsic or 'model' pK_a _values for all protein titratable groups [5]. However, in real protein-solvent systems, interactions between a residue and its environment will cause the titratable group's pK_a _value to deviate from that of the model. Hence the intrinsic pK_a _value, pK_Model_, combined with the environmental perturbation, ΔpK_a_, describes the real pK_a _value of a group [6-9].

**Table 1:** Model pK_a _values for all protein basic and acidic titratable groups. See reference 5.

		**No. in Study**		
**Titratable Group**	**pK_**model **_Value**	**LARGE**	**SMALL**	Mean pK_**exp **_Value
N-Termini	7.5	-	-	-
C-Termini	3.8	-	-	-
Arg	12	1	1	-
Asp	4	143	112	3.5
Cys	9.5	11	4	6.6
Glu	4.4	126	105	4.3
His	6.3	130	24	6.4
Lys	10.4	57	23	9.6
Tyr	10	26	16	9.5

*pK*_*a *_= *pK*_*Model *_+ Δ*pK*_*a *_      (2)

The pK_a _shift caused by the environment is not easily quantified. This is especially true of ionizable residues within protein active sites as they often have markedly higher or lower values than the intrinsic pK_a _[5]. The three main factors that contribute towards environmental perturbation of the pK_a _value are inter-molecular hydrogen bonding, the desolvation effect and Coulombic interactions. Previous studies have identified hydrogen bonding as the most important determinant of pK_a _values [6]. The hydrogen bonding strength is both distance and angle dependent and therefore the extent of the perturbation is heavily dependent on the position of the interacting residues relative to each other. This is less of a factor with side chain hydrogen bonds than with main chain as the former is more flexible and therefore more likely to adopt an optimal orientation for hydrogen bond interactions. The desolvation effect is also important; this describes the energy that is required to move a group from a state of full solvation to a position within the folded protein. Desolvation effects within the protein interior preferentially increases the energies of the negatively-charged, base forms, which will increase the pK_a _value, while in the case of His, Lys and Arg, the desolvation preferentially increases the energy of the positively-charged, acid forms, which will decrease the pK_a _values. The extent of the shift is dependent on the degree to which the group is buried within the protein. The third of the major factors, which may cause a pK_a _shift, are Coulombic interactions between ionizable groups. The pair-wise interactions are dependent on the charges of the respective groups, but also on their location as only residues that are buried produce significant charge-charge interactions.

It is possible to predict the pK_a _value of a given protein residue from three-dimensional structural data. The pK_a _shift may be calculated from the difference in energy between the group's charged and neutral forms and added to the pK_model _value to estimate its true value. Several different algorithms have been developed to generate predicted pK_a _values based on structural data.

The majority of papers which have assessed the reliability of pK_a _predictive algorithms have only examined a limited number of proteins, making an evaluation of their accuracy very difficult [6,10-13]. The largest of these [13] looked at 260 experimental pK_a _values taken from 41 proteins. Here we use a large pK_a _dataset of 100 proteins, which is more than double that of the most extensive previous paper [13], to analyse the predictive capabilities of the MCCE [14,15], MEAD [16], PROPKA [17] and UHBD [18] programs. The programs differ in their methodology and we assessed the merits of each. An enhanced approach to the problem of pK_a _prediction is proposed.

## Results and discussion

Technical difficulties with the UHBD program, due to errors in the protonation of histidine residues, prevented the successful processing of all 100 proteins; in total only 43 could be completed. This lead to the creation of two separate datasets, the Large dataset (containing 492 residues and excluding the UHBD program) and the Small Dataset (containing 280 residues and including the UHBD program). Several of the programs produced outliers, in some cases outside of the physically possible pH 0–14 range. Outliers were removed from the dataset using a variety of different parameters to see the effect upon the overall accuracy of prediction Datasets were generated where all predicted values were lesser or greater than the intrinsic values by 3, 5, 7 and 10 pH units were removed as well a dataset where only physically possible values were included. This data is presented in Table 2 and Table 3. It may been seen from the data that in general the best results were obtained by using values within a range of 5 pH units. Using those parameters, 89 residues were removed from the Large dataset leaving 403 and 39 residues were removed from the Small dataset leaving 241 residues. It is unlikely that the pKa of a titratable residues can deviate more than 5 pH units from the residue's intrinsic value (see Table 1).

**Table 2:** Overview of the prediction accuracy of the Large Dataset (404 Residues) I. The table shows the RMSD values for each of the residues from the whole dataset and the dataset following removal of non-physical values and of all outliers outside of a range of 3, 5, 7 and 10 pH units. Figures marked in bold indicate significant results (*P *= 0.05).

	**AMBER COMPLETE**	**TRUE**	**WITHIN10**	**WITHIN7**	**WITHIN5**	**WITHIN3**	**PARSE COMPLETE**	**TRUE**	**WITHIN10**	**WITHIN7**	**WITHIN5**	**WITHIN3**
**ARG**	*	*	*	*	*	*	*	*	*	*	*	*
**ASP**	2.698	1.928	2.513	1.495	1.354	1.273	3.743	1.729	2.251	1.837	1.25	1.106
**CYS**	*	*	*	*	*	*	*	*	*	*	*	*
**GLU**	1.885	1.466	1.616	1.62	1.42	1.026	3.175	1.48	1.841	1.681	1.442	1.034
**HIS**	3.691	2.171	3.016	2.281	2.022	1.417	3.691	2.122	2.997	2.293	1.993	1.465
**LYS**	1.244	1.263	1.263	1.263	1.122	0.84	25.78	1.162	1.162	1.162	0.991	0.741
**TYR**	2.739	2.195	2.195	2.195	1.939	1.516	3.163	2.143	2.143	2.143	1.882	1.481

	**MCCE COMPLETE**	**TRUE**	**WITHIN10**	**WITHIN7**	**WITHIN5**	**WITHIN3**	**PROPKA COMPLETE**	**TRUE**	**WITHIN10**	**WITHIN7**	**WITHIN5**	**WITHIN3**

**ARG**	*	*	*	*	*	*	*	*	*	*	*	*
**ASP**	2.024	1.774	2.032	1.79	1.534	1.242	1.301	1.041	1.313	1.301	**0.934**	0.806
**CYS**	*	*	*	*	*	*	*	*	*	*	*	*
**GLU**	1.642	1.255	1.335	1.296	1.259	0.97	1.011	0.851	0.994	0.997	**0.849**	0.611
**HIS**	4.503	1.736	2.598	2.104	**1.522**	1.718	1.865	1.586	1.819	1.631	**1.530**	1.551
**LYS**	1.137	1.125	1.125	1.125	1.005	1.129	0.417	0.412	0.412	0.412	**2.600**	0.423
**TYR**	5.426	2.643	2.634	2.643	1.668	1.593	2.225	1.551	1.551	1.551	**1.001**	1.049

Outliers

COMPLETE 0 removed

TRUE 52 removed

WITHIN 10 23 removed

WITHIN 7 27 removed

WITHIN 5 89 removed

WITHIN 3 110 removed

**Table 3:** Overview of the prediction accuracy of the Small Dataset (242 Residues) I. The table shows the RMSD values for each of the residues from the whole dataset and the dataset following removal of non-physical values and of all outliers outside of a range of 3, 5, 7 and 10 pH units. Figures marked in bold indicate significant results (*P = 0.05*).

	**AMBER COMPLETE**	**TRUE**	**WITHIN10**	**WITHIN7**	**WITHIN5**	**WITHIN3**	**PARSE COMPLETE**	**TRUE**	**WITHIN10**	**WITHIN7**	**WITHIN5**	**WITHIN3**
**ARG**	*	*	*	*	*	*	*	*	*	*	*	*
**ASP**	2.078	1.787	2.079	2.077	1.691	1.781	3.774	1.582	2.142	1.912	1.53	1.267
**CYS**	*	*	*	*	*		*	*	*	*	*	
**GLU**	1.641	1.416	1.603	1.603	1.294	1.047	3.041	1.474	1.71	1.71	1.334	1.051
**HIS**	2.786	1.689	1.689	1.689	1.347	1.118	2.736	1.809	1.81	1.81	1.488	1.402
**LYS**	1.291	1.29	1.291	1.291	1.291	1.291	1.278	1.278	1.278	1.278	1.278	1.278
**TYR**	2.06	1.297	1.297	1.297	1.933	0.766	2.368	1.262	1.262	1.262	1.871	0.792

	**MCCE COMPLETE**	**TRUE**	**WITHIN10**	**WITHIN7**	**WITHIN5**	**WITHIN3**	**UHBD COMPLETE**	**TRUE**	**WITHIN10**	**WITHIN7**	**WITHIN5**	**WITHIN3**

**ARG**	*	*	*	*	*	*	*	*	*	*	*	*
**ASP**	1.915	1.735	1.921	1.731	1.319	1.419	0.95	0.824	0.838	0.842	0.89	0.641
**CYS**	*	*	*	*	*		*	*	*	*	*	
**GLU**	1.575	1.185	1.237	1.237	1.188	0.893	0.508	0.478	0.493	0.493	**0.442**	0.395
**HIS**	1.985	1.584	1.584	1.584	1.056	1.593	0.634	0.453	0.453	0.453	**0.494**	0.428
**LYS**	1.152	1.152	1.152	1.152	1.152	1.152	0.412	0.412	0.412	0.412	0.412	0.412
**TYR**	6.027	1.373	1.373	1.373	1.456	1.419	0.687	0.582	0.582	0.582	0.61	0.631

	**PROPKA COMPLETE**	**TRUE**	**WITHIN10**	**WITHIN7**	**WITHIN5**	**WITHIN3**						

**ARG**	*	*	*	*	*	*						
**ASP**	1.827	1.826	1.806	1.809	**0.879**	0.745						
**CYS**	*	*	*	*	*							
**GLU**	0.987	0.773	0.959	0.959	0.781	0.632						
**HIS**	2.235	2.11	2.11	2.11	1.724	2.172						
**LYS**	0.394	0.394	0.394	0.394	**0.394**	0.394						
**TYR**	1.533	0.992	0.991	0.991	**0.572**	1.011						

Outliers

COMPLETE 0 removed

TRUE 25 removed

WITHIN 10 11 removed

WITHIN 7 13 removed

WITHIN 5 34 removed

WITHIN 3 54 removed

**Figure 1:**
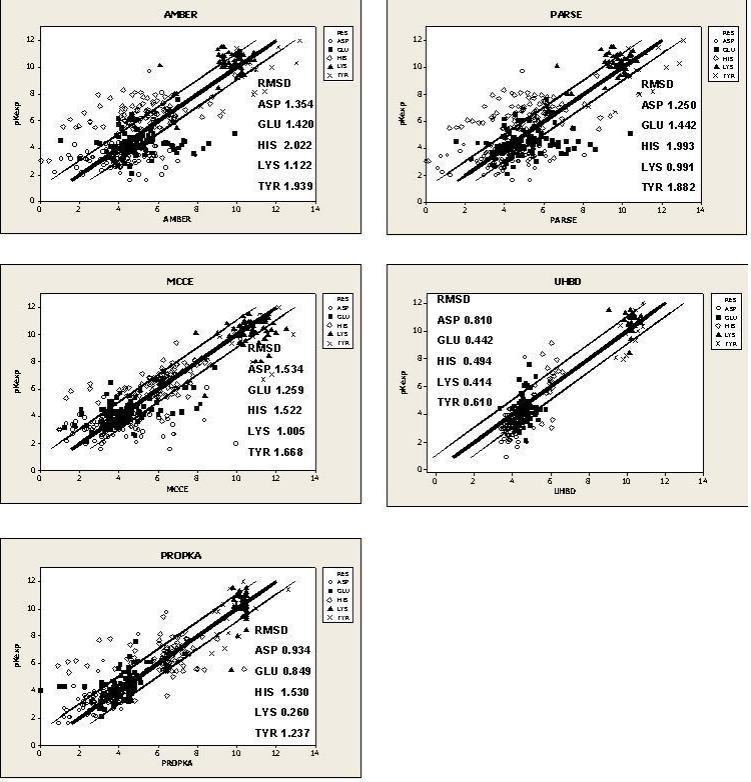
**Correlation plots for the individual programs**. The bold line indicates perfect prediction (pK_pred _= pK_exp_). The outer lines indicate +/- 1 unit from the pK_exp_.

The majority of the outliers in both datasets were generated by the MEAD program, particularly when the PARCE force field was used. Considerably more residues are present within the +/- 1 unit bands for MCCE, UHBD and PROPKA. Thus there is a clear division between the performance of MEAD and that of the other programs. The same trend may be seen in the Root Mean Squared Deviation (RMSD) values (Table 2, 3). PROPKA is more accurate for Asp, Glu, Lys and Tyr with RMSD values of 0.934, 0.849, 0.260 and 1.001 respectively. His is more accurately predicted by MCCE with an RMSD of 1.522. With respect to the Small dataset in Table 3, PROPKA is the best predictor for all residues except Glu and His, where UHBD performs best: RMSD of 0.442 and 0.494 respectively. The overall accuracy of each program to a level of <0.5 pK_a _units is 27% AMBER, 34% PARSE, 42% MCCE, 40% UHBD (242 dataset) and 48% PROPKA. When the error range is increased to <1 unit, the difference between the programs is more distinct: 56% AMBER, 56% PARSE, 71% MCCE, 67% UHBD (Small dataset) and 81% PROPKA (Table 4, 5). Scatter plots for each program are shown in Figure 1.

**Table 4:** Overview of the prediction accuracy of the Large Dataset (404 Residues) II Three tables show the accuracy of the predictions to the measured pK_exp _within the ranges of <2 to <0.5. This is taken as the number of residues predicted within each range. Figure marked in bold indicate significant results (*P = 0.05*).

**TOTAL (404)**								
	**AMBER**		**PARSE**		**MCCE**		**PROPKA**	
		**%**		**%**		**%**		**%**
<2	322	80	320	79	357	88	371	**92**
<1.5	283	70	282	70	328	81	354	**88**
<1	213	53	221	55	283	70	317	**78**
<0.5	108	27	139	34	168	42	195	**48**

**SURFACE (337)**								
	**AMBER**		**PARSE**		**MCCE**		**PROPKA**	
		**%**		**%**		**%**		**%**

<2	285	85	284	84	311	92	329	**98**
<1.5	255	76	253	75	290	86	317	**94**
<1	196	58	200	59	248	74	285	**85**
<0.5	98	29	132	39	151	45	184	**55**

**BURIED (66)**								
	**AMBER**		**PARSE**		**MCCE**		**PROPKA**	
		**%**		**%**		**%**		**%**

<2	37	56	36	55	46	**70**	42	64
<1.5	28	42	29	44	38	**58**	37	56
<1	17	26	21	32	35	**53**	32	48
<0.5	10	15	7	11	17	**26**	11	17

**Table 5:** Overview of the prediction accuracy of the Small Dataset (242 Residues) II. Three tables show the accuracy of the predictions to the measured pK_exp_. This is taken as the number of residues predicted within each range. Figures marked in bold indicate significant results (*P = 0.05*).

**TOTAL (242)**										
	**AMBER**		**PARSE**		**MCCE**		**UHBD**		**PROPKA**	
		**%**		**%**		**%**		**%**		**%**
<2	195	81	191	79	216	89	225	93	230	95
<1.5	174	72	171	71	201	83	209	86	220	91
<1	131	54	135	56	172	71	161	67	195	81
<0.5	63	26	87	36	110	45	97	40	125	52

**SURFACE (209)**										
	**AMBER**		**PARSE**		**MCCE**		**UHBD**		**PROPKA**	
		**%**		**%**		**%**		**%**		**%**

<2	179	86	176	84	193	92	199	95	205	**98**
<1.5	163	78	159	76	181	87	186	89	199	**95**
<1	126	60	125	60	156	75	152	73	181	**87**
<0.5	60	29	83	40	100	48	94	45	122	**58**

**BURIED (33)**										
	**AMBER**		**PARSE**		**MCCE**		**UHBD**		**PROPKA**	
		**%**		**%**		**%**		**%**		**%**

<2	16	48	15	45	23	70	26	**79**	25	76
<1.5	11	33	12	36	20	61	23	**70**	21	64
<1	5	15	10	30	16	**48**	9	27	14	42
<0.5	3	9	4	12	16	**30**	3	9	3	9

From a previous study [19], 39 carboxyl residues found within protein active sites were selected. These are shown in Table 6[20-30]. The 27 Asp and 12 Glu residues have experimental values that differ from the model pK_a _value by at least 1 unit. Values for Asp and Glu range from 2.0 – 9.9 and 2.1 – 6.7 respectively. PROPKA and UHBD are distinct within the <0.5 and <1 unit error bands (Table 7), with PROPKA performing best with an accuracy of 66.67% within the <1 unit band. However a large discrepancy exists between the <1 and <0.5 bands for all of the programs, with an approximate 50% drop in accuracy. For the Asp residues, PROPKA predicts far better than the other programs, with values of 37.04%, compared to 18.52% for MCCE and 0% for UHBD at the <0.5 level. However, for Glu residues program performance is closer, with values of 25 % for PARSE, MCCE and UHBD and 33% for AMBER and PROPKA at the <0.5 level. When the error level is extended to <1, PROPKA is far better, with a value of 83.33% compared to its nearest rivals UHBD, AMBER and PARSE with values of 50%. PROPKA shows an accuracy of 85% to within 1 pK_a _unit for surface residues, whereas the same accuracy is limited to 53% with the MCCE program for buried residues, a considerable reduction. The accuracy values obtained for MCCE were also comparable with those that recently appeared on the program's website, which show an RMSD of 0.77 and an accuracy range of 98% within the <2 pH unit range and 84% accuracy within the <1 pH unit range [31].

**Table 6:** Carboxyl sites of interest. B = Buried, S = Surface. Figures marked in bold indicate predictions >2 units from the pK_exp_.

**PDB**	**RESIDUE**	**LOCATION**	**AMBER**	**PARSE**	**MCCE**	**UHBD**	**PROPKA**	**pKexp**
1A2P^20^	ASP-101	S	**6.19**	3.56	2.31	3.75	1.20	2.00
	ASP-93	S	**-0.64**	**-1.38**	1.00	3.92	0.69	2.00
	ASP-54	S	0.74	0.82	1.34	3.57	2.70	2.00
	GLU-73	B	**4.66**	4.10	2.37	**4.68**	3.11	2.10
1A91^21^	ASP-7	S	3.99	**20.29**	4.17	4.04	3.87	5.60
	ASP-44	S	6.00	**20.22**	5.55	4.69	4.19	5.60
	ASP-61	S	5.52	**26.20**	5.01	**4.33**	**4.01**	7.00
	GLU-2	S	4.14	7.45	4.53	4.45	4.50	5.50
	GLU-37	S	5.15	**-20.96**	4.27	4.66	4.32	5.50
1BEO^22^	ASP-21	S	**6.38**	**5.52**	3.11	3.75	1.35	2.50
	ASP-30	S	3.56	3.80	4.38	4.00	2.64	2.50
	ASP-72	S	3.84	3.95	3.69	4.34	3.30	2.60
1DE3^23^	GLU-96	B	**9.88**	**10.41**	3.53	5.70	4.10	5.10
	GLU-115	S	5.19	5.23	3.81	4.45	4.50	4.90
1KXI^24^	ASP-59	S	3.13	3.90	2.33	4.20	2.49	2.30
1LZ3^25^	ASP-18	S	3.79	3.94	**6.77**	4.05	3.19	2.70
	ASP-48	S	3.57	3.17	**5.15**	3.99	2.51	2.50
	ASP-66	S	0.23	**-0.31**	**12.38**	3.07	1.19	2.00
	ASP-87	S	4.06	3.96	**4.89**	3.89	2.17	2.10
	GLU-7	S	3.92	3.44	**4.73**	4.36	3.01	2.70
	GLU-35	B	4.92	5.23	7.80	4.78	5.40	6.10
1RNZ^26^	ASP-14	B	**9.08**	**7.86**	3.51	**4.89**	**-0.62**	2.00
	GLU-2	B	**-1.52**	**-1.48**	1.44	**5.03**	2.66	2.80
1TRS^27^	ASP-26	B	6.18	**4.38**	7.84	**4.64**	**4.96**	8.10
	GLU-6	S	3.91	3.92	4.54	4.44	4.50	4.90
	GLU-68	S	4.27	4.24	4.59	4.55	4.57	5.10
1TRW^27^	ASP-26	B	8.23	**5.24**	8.63	**4.83**	**5.62**	9.90
	GLU-68	S	5.07	5.33	3.55	4.88	4.34	4.90
1XNB^28^	ASP-11	S	1.83	0.57	3.44	3.82	1.99	2.50
	ASP-83	B	**6.29**	**7.89**	6.35	**4.28**	1.36	2.00
	ASP-101	B	**5.01**	2.94	**9.96**	**4.28**	1.50	2.00
	ASP-106	S	**8.72**	**8.94**	3.18	**4.98**	3.02	2.70
	GLU-172	B	6.62	6.42	5.94	5.22	7.32	6.70
2OVO^29^	ASP-7	S	4.05	4.01	**6.25**	3.72	2.51	2.50
	ASP-27	S	2.08	2.32	2.77	3.77	2.39	2.50
2RN2^30^	ASP-10	B	**4.01**	**3.38**	**8.47**	**3.83**	6.99	6.10
	ASP-70	B	4.11	3.55	3.15	3.50	4.10	2.60
	ASP-102	B	**7.33**	**6.77**	3.00	3.40	0.13	2.00
	ASP-148	B	-1.10	**-1.31**	0.55	3.79	**-0.79**	2.00

**Table 7:** Accuracy of prediction for the carboxyl sites. The accuracy was tested to the <2 to <0.5 ranges. The individual accuracy of the residues is given in the bottom two tables. Figures marked in bold indicate the greatest accuracy.

**TOTAL (242)**										
	**AMBER**		**PARSE**		**MCCE**		**UHBD**		**PROPKA**	
		**%**		**%**		**%**		**%**		**%**
<2	26	66.67	20	51.28	30	76.92	30	76.92	34	**87.18**
<1.5	19	48.72	15	38.46	25	64.10	20	51.28	32	**82.05**
<1	10	25.64	10	25.64	17	43.59	8	20.51	26	**66.67**
<0.5	6	15.38	4	10.26	8	20.51	3	7.69	14	**35.90**

**SURFACE (209)**										
	**AMBER**		**PARSE**		**MCCE**		**UHBD**		**PROPKA**	
		**%**		**%**		**%**		**%**		**%**

<2	17	62.96	13	48.15	10	70.37	19	70.37	22	**81.48**
<1.5	10	37.04	8	29.63	16	59.26	11	40.74	20	**74.07**
<1	4	14.81	4	14.81	12	44.44	2	7.41	16	**59.26**
<0.5	2	7.41	1	3.70	5	18.52	0	0.00	10	**37.04**

**BURIED (33)**										
	**AMBER**		**PARSE**		**MCCE**		**UHBD**		**PROPKA**	
		**%**		**%**		**%**		**%**		**%**

<2	9	75.00	9	75.00	11	91.67	11	91.67	12	**100.00**
<1.5	9	75.00	7	58.33	9	75.00	9	75.00	12	**100.00**
<1	6	50.00	6	50.00	5	41.67	6	50.00	10	**83.33**
<0.5	4	**33.00**	3	25.00	3	25.00	3	25.00	4	**33.33**

Given the capacity of the predictive programs to under or over predict the true pK_a _value (see Discussion), the possibility of using a consensus approach to integrate the various programs was investigated. Using the Small dataset, combinations of the prediction values were calculated and the accuracy tested as before. From this dataset, 25 combinations (Table 8) were tried. One, UHBD + PROPKA, leads to improvements in all residues except histidine. The His RMSD value of 0.955, from this combination, is far better than all of the programs except UHBD, which has a value of 0.494. The overall accuracy of this combination was not a surprise due to the individual performance of each program. A further attempt to integrate the programs was made by using Partial Least Squared (PLS) regression. The PLS model generated had a correlation coefficient (*r*^*2*^) of 0.9 and a cross-validated correlation coefficient (*q*^*2*^) of 0.89. The resulting equations were applied to the Small dataset and the accuracy results are shown in Table 9. The accuracy improved greatly in the <0.5 range to almost 58%, significantly greater than either the other programs run individually or the combination method (UHBD + PROPKA). Again, a disparity between the predictive capabilities of MEAD and the other programs may be seen. AMBER and PARSE have coefficients of -0.03129 and -0.0001836 respectively, which are comparatively small compared to the other coefficients (0.239, 0.4282 and 0.4108 for MCCE, UHBD and PROPKA respectively). The results above would indicate a more significant relationship between the PROPKA and UHBD predictions and the experimental pK_a _values. This data fits with the trends seen in the other analysis. However, multiple linear regression is not an ideal way to increase predictive accuracy as combining programs will cause the propagation of experimental errors within a given dataset.

**Table 8:** Overview of the combination methods (242 Residues). The residue RMSD values are given for all of the 25 combinations consisting of AMBER (A), PARSE (P), MCCE (M), UHBD (U) and PROPKA (P). Figures marked in bold indicate an improvement while the asterisk indicates the best score.

	**A + P**	**A +M**	**A + U**	**A + PR**	**P + M**
**ASP**	1.556	1.245	1.174	**0.837**	1.161
**GLU**	1.301	1.012	0.816	0.676	1.026
**HIS**	1.331	0.631	0.827	1.000	0.727
**LYS**	1.281	0.763	0.742	0.646	0.793
**TYR**	1.899	1.512	1.238	1.028	1.502

	**P + U**	**P + PR**	**M + U**	**M + PR**	**U + PR**

**ASP**	1.074	**0.786**	0.910	**0.818**	**0.596**
**GLU**	0.834	0.690	0.660	0.766	**0.393**
**HIS**	0.812	0.987	0.566	1.289	0.955
**LYS**	0.742	0.647	0.713	0.755	**0.366**
**TYR**	1.212	1.016	0.974	0.956	**0.466**

	**A + P + M**	**A + P + U**	**A + P + PR**	**A + M + U**	**A + M + PR**

**ASP**	1.262	1.242	1.000	1.038	**0.837**
**GLU**	1.064	0.973	0.844	0.761	0.714
**HIS**	0.792	0.954	0.942	0.520	0.839
**LYS**	0.868	0.913	0.846	0.615	0.590
**TYR**	1.607	1.446	1.300	1.195	1.097

	**A + U + PR**	**P + M + U**	**P + M + PR**	**M + U + PR**	**A + P + M + U**

**ASP**	**0.773**	0.972	**0.795**	**0.681**	1.101
**GLU**	0.539	0.770	0.720	0.529	0.870
**HIS**	0.771	0.523	0.840	0.897	0.646
**LYS**	0.518	0.634	0.610	0.597	0.718
**TYR**	0.869	1.188	1.098	0.782	1.345

	**A + P + M + PR**	**A + P + U + PR**	**A + M + U + PR**	**P + M + U + PR**	**A + P + M + U + PR**

**ASP**	0.929	0.905	**0.779**	**0.738**	**0.866**
**GLU**	0.798	0.706	0.588	0.592	0.690
**HIS**	0.754	0.771	0.690	0.668	0.650
**LYS**	0.683	0.689	0.525	0.540	0.605
**TYR**	1.256	1.112	0.959	0.959	1.115

**Table 9:** Accuracy of the multiple regression. The accuracy is given as the number of predictions within a range of the pK_exp_. For comparison the UHBD + PROPKA combination is added. Figures marked in bold indicate improvements.

**TOTAL (242)**				
	**REGRESSION**		**UHBD + PROPKA**	
		%		%
<2	234	96.69	233	96
<1.5	228	**94.21**	226	93
<1	205	**84.71**	197	81
<0.5	140	**57.85**	124	51

The disparity between the predictive capabilities of the program relates to the algorithm that used for the calculation. MEAD, UHBD and MCCE are all based upon an electrostatic continuum model that solves the linearised Poisson-Boltzmann equation numerically [32,33]. The electrostatic potential φ(*r*)can be calculated by the Poisson-Boltzmann equation:

∇ ε (*r*)∇ φ (*r*) - κ^2 ^(*r*)ε (*r*)φ (*r*) = -4πρ (*r*)       (3)

Whereε is the dielectric constant, *r *is the position vector, *Φ *is the electrostatic potential, ρ is the charge distribution and κ is a parameter that represents the effect of mobile ions in solution. All three programs work on the assumption that the major determinant of the pK_a _shift from the model values are the electrostatic effects of burying titratable groups in low dielectric medium. A model of the macromolecule-solvent system is used with dielectric constants of 80 for the solvent and 4 for the protein. The details of the atomic structure are incorporated into the placement of charges and dielectric boundaries. The calculation accounts for the desolvation energy, the titratable group's interaction with partial charges and the group's interaction with other titratable groups in the protein. MEAD consistently performs more poorly than the other two Poisson-Boltzmann-based programs. This may be because, in addition to the basic calculation, UHBD and MCCE also incorporate a Monte Carlo function to sample the multiple conformations of each titratable site. The Monte Carlo method achieves convergence by random sampling of side chain conformers. This allows the MCCE and UHBD programs to make a more realistic calculation of the charge-charge interactions than MEAD. The RMSD values of the two MEAD data sets – PARSE and AMBER – are comparable, both producing similar RMSD values and numbers of outliers. However, Table 2 shows that the PARSE force field generates outliers that deviate much further from the experimentally-determined values than those of AMBER. Although the parameters of the two force fields are similar; the atomic radii of the hydrogens for PARSE are slightly larger which may have created inaccurate charge-charge interactions that have increased the calculated pK_a _value (this would also account for the program's propensity to generate outliers). This is especially noticeable for Lys where the respective RMSD values of AMBER and PARSE are 1.2 and 25.8.

Although the PROPKA and MCCE programs are of comparable accuracy, the data suggests that the former tends to under-predict pK_a _values whilst the latter over-predicts them (Figure 1). This observation may reflect the different approaches towards the calculation of the pK_a _value in the two programs. PROPKA [17] takes a different approach to the other three programs, calculating the pK_a _shift by using empirical rules that incorporate effects from hydrogen bonds, desolvation and Coulombic interactions. The extent of the pK_a _shift caused by hydrogen bonding is proportional to the number of hydrogen bonds formed by the titratable group [19]. The desolvation effect is calculated from the solvent accessible surface and the 'depth of burial' (the distance of the group from the protein surface). Lastly, the strength of the Coulombic charge-charge interactions is dependent on the distance between the charges and on the state of the surrounding ionizable residues. This process, however, is only applied to buried pairs of ionizable residues. Therefore PROPKA's tendency to under-predict pK_a _values may be caused by the program's emphasis on the dominance of hydrogen bonding in determining the extent of the shift. Hydrogen bonds have the effect of lowering pK_a _values [34] and the predicted values may reflect that. Conversely, MCCE's tendency to over-predict may be the result of charge-charge interaction forcing an increase in the pK_a _value. The majority of the over-predicted values are surface residues and, unlike PROPKA, the MCCE program does not take into account the lessened effects of charge-charge interactions when the respective residues are not buried within the protein interior. Consequently, PROPKA and MCCE tend to be more accurate for surface and buried residues respectively.

Side chains located at active sites are of particular interest as they often have unusually high or low pK_a _values. In some instances, the electrostatic charge of the active site can be radically different from that the rest of the protein as a means to 'steer' a ligand towards the binding cleft [35]. For a program to work as an effective pK_a _prediction tool it must be able to predict unusual pK_a _values accurately. Generally speaking, the accuracy of prediction decreased the further the measured pK_a _value was from the side chain's intrinsic pK_a_. Again, PROPKA proved to be the most consistent of the programs. This is not surprising as the design of the model and assignment of parameters were based upon a large dataset of carboxyl pK_a _values. Overall, the active site data was encouraging at the <1 unit level. However, once reduced to <0.5, the accuracy of all of the programs decreased. This highlights a key area for the development of new models and programs.

**Figure 2:**
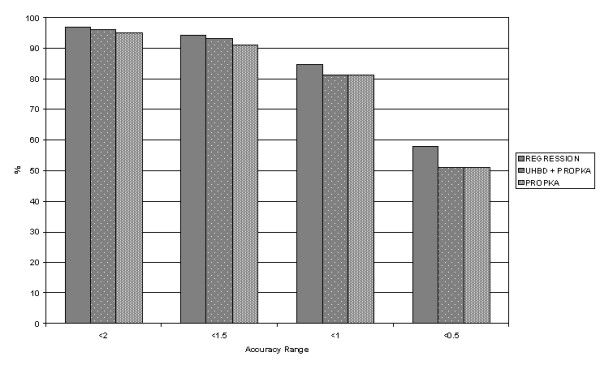
**Comparative performance of the prediction methods**. The accuracy ranges (0.5 – 2) apply to the deviation from the measured pK_a _value. The percentage score represents the number of residues predicted in each range.

An interesting correlation is seen with respect to the regression coefficients and the general performance of the programs. Coefficients are generally an indicator of the relative importance of the contributing terms in a regression equation. The comparative performance of PROPKA, the combination methods and the regression model is seen in Figure 2. PROPKA is equally effective as these additional methods, although the regression data performs better than the best combination. A Molecular Dynamics simulation of one of the proteins from the dataset (Barnase wild type ribonuclease (*pdb *code: 1A2P)) showed a standard deviation of ± 1.4 for the pK_a _value over a one-nanosecond period. This indicates that a dynamic structure has a large capacity for extreme pK_a _shifts. This suggests that any accurate prediction pK_a _method would need to incorporate conformational variability into the algorithm.

## Conclusion

PROPKA is the most accurate method for all residues except Glu and His, where it is narrowly surpassed by UHBD and MCCE, respectively. Furthermore, the program also produces by far the best values for surface residues, most likely by taking sufficient account of hydrogen bonding. However, MCCE predicts buried residues far better than PROPKA, possibly by a more accurate evaluation of the charge-charge interaction with the conformers optimised by the Monte Carlo procedure. It must be noted that in all cases, the prediction of the buried residues is less accurate than for surface residues, indicating it is easier to calculate the interaction of a solvated or partially solvated residue than one densely packed within the protein interior. Overall, the best standalone program is PROPKA, which also produced the fewest outliers and is computationally much faster than the other programs. What the program lacks is a capacity to fully explore the conformational space available to the protein, which may ultimately limit its capacity to predict pK_a _value. The reliability of the predictive programs tends to vary with both the residue type and its spatial location. For glutamic acid residues, UHBD produced the best results while for Histidine and for all buried residues, MCCE performed well. The comparatively poor prediction of the 'unusual' pK_a _values by all of the programs was disappointing. Their ability to only predict a third of the residues to a high degree of accuracy highlights an area requiring further development. The variation in pK_a _values observed in our molecular dynamics simulation strongly suggests a complete sampling of conformations accessible to protein structures may be useful in creating accurate predictive software.

## Methods

100 proteins for which pK_a _values had been determined experimentally were taken from PPD, a database of protein ionization constants [36,37]. The full list of the *pdb *files comprising the dataset is included as an additional file [See PDB codes]. A wide range of both protein size and function was represented in the dataset. The protein structures were taken from the RCSB protein data bank [38]. In order to run the MEAD program, *pdb *files were protonated by using the *leap *program and the AMBER 94 force field (subsequent versions of the force field proved to be incompatible) and changed into *pqr *format using the online PDB2PQR converter [39,40]. Separate sets of files were created based on the AMBER99 and PARSE force fields. MEAD and UHBD were run on an IBM Blade Center Cluster, which consists of 5 Blade Centers containing 67 Dual Xeon (3.06Ghz, 1Gb) Blades. The MCCE calculations were carried out on an SG Octane. The majority of the *pdb *files did not need any modification. However, 1D3K, 1GU8, 1HRH and 1DRH were protonated with the *leap *program and the AMBER 03 force field in order to remove inconsistencies in the *pdb *files. Additionally, 1DUK, 1NFN and 2CI2 underwent minimization with *sander *using a steepest descent method that continued for 20,000 1 fs time steps or until the root mean square deviation between successive time-steps had fallen below 0.01Å in order to eliminate steric clashes. The PROPKA program was run online from its server [41]; no modification was required to run the files. Values for all Asp, Glu, His, Tyr, Lys residues were predicted. Arg was excluded from the calculation due to lack of experimental data. Arginines's high pK_a _precludes establishing a titratable curve as the protein denatures at high pH. Cys was also excluded from the calculations due to a lack of experimental data.

The resultant data was also analysed using the Partial Least Squares (PLS) method. PLS is an extension of Multiple Linear Regression (MLR) that where a set of coefficients are developed from dependent variables, in this case the pK_a _prediction values, by comparison with the independent variables, the experimental pK_a _values. The PLS analysis was performed using the program GOLPE (Generating Optimal Linear PLS Estimations)[42].

## Authors' contributions

MND formatted the data carried out the calculations for all of the pK_a _programs mentioned. CPT assembled the data set and carried out statistical analysis on the output of the pK_a _programs. DSM supervised the pK_a _calculations using the MEAD and UHBD programs at Birkbeck College. DRF instigated and supervised the entire project. MND, CPT and DRF drafted the manuscript. All authors have read and accepted the manuscript.

## Supplementary Material

Additional File 1 PDB codes, a full list of the pdb codes for the three-dimensional structures comprising the dataset. Click here for file
