# Supplementary material for: Benchmarking pKa prediction
Source: BMC Biochem. 2006 Jun 2;7:18. doi: 10.1186/1471-2091-7-18 (PMC1513386; doi:10.1186/1471-2091-7-18)
Supplement: Additional File 1 — PDB codes, a full list of the pdb codes for the three-dimensional structures comprising the dataset. [file 1471-2091-7-18-S1.doc]

# PDB codes

1A2P

1AG1

1ARP

1B2V

1BEO

1BQK

1BVC

1BXK

1C3W

1CB4

1CI1

1CMF

1D3K

1DE3

1DG9

1DIV

1DRH

1DUI

1ERT

1EY0

1FKS

1FW4

1GDC

1GS9

1GU8

1GU9

1GUK

1GYM

1HNG

1HPX

1ID2

1IGD

1J8Q

1KXI

1L63

1LZ1

1LZ3

1MUT

1OH0

1PGA

1PLB

1PNT

1POH

1PPO

1RGA

1RNZ

1SBT

1SSO

1STN

1TDE

1TRS

1TRW

1UBQ

1XNB

1YMB

2A0B

2CI2

2CPL

2LZM

2LZT

2OVO

2RN2

2SNM

3RN3

3RNT

3SSI

4ICB

4MBN

6GST

6LYZ

7FD1

7RSA

9RNT

1CDC

1DPO

1SBC

1NFN

1GSD

1GUH

1QH7

1DPG

1KB9

1NFO

1WB8

2TRX

1ISA

1NAI

1A91

1B0D

1CF3

1SPU

1AXT

1ERU

1EWD

1DSB

1MG5

1EWE

1ADO

1P2P

1MEG
